# Evaluating the Correlation Between Prosthodontic Interventions and Chest Infections: A Comprehensive Research Analysis

**DOI:** 10.7759/cureus.66208

**Published:** 2024-08-05

**Authors:** Muhammad Abdul Muqeet, Muhammad Uzair Riaz, Summaiya Qaisar, Naveed Ahmad, Amna Nisar, Syeda Sameen Zehra Rizvi

**Affiliations:** 1 Department of Prosthodontics, Dental College, Heavy Industries Taxila Education City Institute of Medical Sciences (HITEC-IMS), Taxilla, PAK; 2 Department of Prosthodontics, De'Montmorency College of Dentistry, Lahore, PAK; 3 Department of Prosthodontics, School of Dentistry, Islamabad, PAK

**Keywords:** tooth restoration, oral health, dental patient-reported outcomes, chest infections, prosthodontic interventions

## Abstract

Background: There is growing interest in the relationship between prosthodontic therapies and outcomes related to systemic health, particularly respiratory infections. Respiratory infections are a leading cause of global morbidity and mortality, particularly among vulnerable populations such as immunocompromised individuals. The World Health Organization reports that lower respiratory infections are among the top causes of death worldwide, underscoring the importance of understanding their potential link to prosthodontic procedures. Dental operations, such as prosthodontic therapies, may alter the mouth flora and thus affect respiratory health.

Objective: This research aimed to investigate the relationship between prosthodontic procedures and chest infections.

Methodology: This research was an observational prospective cohort study conducted from January 2023 to December 2023 at the Pakistan Institute of Medical Sciences (PIMS) located in Islamabad, Pakistan. One hundred thirty individuals in the cohort, who were at least 18 years old, had a range of prosthodontic procedures, such as total edentulous solutions and tooth restoration. Electronic health data were used for participant selection to minimize selection bias and guarantee diverse representation. Comprehensive evaluations of cardiovascular health, immunological state, pulmonary function, and medical histories were all part of the data-gathering process. Structured questionnaires and interviews were also used to get patient feedback. Using SPSS Statistics software (version 27; IBM Corp., Armonk, NY), statistical analysis was performed to examine the relationships between prosthodontic treatments and chest infections using descriptive statistics and logistic regression.

Results: PIMS hosted 130 participants in this research, which found that 29 patients, or 22% of the total, had chest infections after surgery. Chest infection rates were 15.15% (five out of 33) in the 18-39 age group, 21.21% (14 out of 66) in the 40-59 age group, and 32.26% (10 out of 31) in the 60+ age group, according to age-specific analysis. Complete edentulous solutions (38 cases, 29.23%) and tooth restoration (55 cases, 42.31%) were the most frequently performed procedures. Compared to tooth restoration (n=15; 55.17%), complete edentulous solutions (n=8; 27.59%) had increased infection risks, according to logistic regression. Following prosthodontic procedures, respiratory health measures improved: respiratory rate dropped to 17.8/min, oxygen saturation rose to 98.1%, and frequency of coughing fell to 1.9/day.

Conclusion: This research highlights the need for careful post-operative respiratory surveillance by elucidating the strong associations between prosthodontic procedures and chest infections.

## Introduction

Academics and healthcare professionals have been paying more and more attention to the connection between systemic disorders and dental health [1]. The possible relationship between prosthodontic procedures and respiratory infections, especially chest infections, is one particularly fascinating field of research [2]. In order to restore oral function and aesthetics, prosthodontic interventions - which include the use of dental prostheses and dental implants - are often performed [3]. Still under research is their effect on more general health outcomes, such as respiratory infections [4].

Numerous bacteria may be found in the mouth cavity, and some of them can aspirate into the lower respiratory system and cause illnesses [5]. The microbial ecology of the oral cavity and, therefore, the risk of respiratory infections, may be influenced by dental treatments, particularly those that involve the manipulation of oral tissues and the introduction of foreign materials (such as dental prostheses) [6]. It is important to comprehend the possible connection between chest infections and prosthodontic procedures for a number of reasons. First off, pneumonia and other respiratory infections continue to be a major global source of morbidity and death, particularly in susceptible groups like the elderly and immunocompromised people [7-9]. Second, elucidating this correlation might guide clinical procedures and preventative measures, as the world's population ages and the number of people choosing dental prostheses rises [10].

The specifics of this association are still unclear, despite some research suggesting potential pathways via which dental therapies may affect respiratory health [11]. This link may be significantly shaped by variables including the kind of prosthodontic operation, patient demographics, underlying medical disorders, and post-operative care guidelines [12]. It is crucial to fill up these knowledge gaps with thorough research to provide doctors with evidence-based advice and enhance patient outcomes.

Although the field is becoming more and more interested in this subject, there is still a significant lack of thorough research that systematically assesses the relationship between prosthodontic procedures and chest infections in a variety of patient types and environments. The majority of the existing literature is made up of small-scale research with inconsistent methodology and sometimes contradictory findings, which emphasizes the need for a comprehensive and well-organized study.

Research objective

This research aimed to provide a thorough analysis of the relationship between prosthodontic procedures and chest infections.

## Materials and methods

Study design and settings

The Heavy Industry Taxila (HIT) Hospital, Taxila Cantt, Pakistan, hosted this research from January 2023 to December 2023 for a duration of one year. It used a prospective cohort design to look at the relationship between chest infections and prosthodontic procedures.

Inclusion and exclusion criteria

Participants included 130 individuals aged 18 or above who underwent various prosthodontic procedures, encompassing both tooth restoration and complete edentulous solutions. In order to avoid selection bias and assure variety in prosthodontic procedures, patients were chosen from electronic health data. People with unfinished medical records or a history of respiratory illnesses were among the exclusion criteria.

Sample size and sampling technique

A total of 130 participants were included to ensure robust statistical analysis and sufficient power to identify associations between prosthodontic procedures and chest infections. Participants were selected from electronic health records at HIT Hospital to minimize selection bias and ensure a diverse representation of prosthodontic procedures. This sample size was determined to detect significant associations while adequately considering data quality and variability.

Follow-up procedures

Participants who underwent prosthodontic interventions were monitored over a specified follow-up period to assess post-operative health outcomes, including the incidence of chest infections. Follow-up visits were scheduled at multiple intervals: within <1 month, one to three months, and beyond three months after the intervention. During these visits, participants were evaluated for symptoms and signs of respiratory infections.

Assessment and diagnosis of chest infections

Chest infections were assessed through clinical evaluation and confirmatory diagnostic tests, including sputum culture to identify pathogenic microorganisms and blood tests such as complete blood count (CBC) and C-reactive protein (CRP) levels to assess systemic inflammation or infection. These procedures ensured accurate diagnosis and evaluation of the relationship between prosthodontic procedures and respiratory health outcomes.

Data collection

Data collection involved a comprehensive review of participants' medical histories, focusing on immune system status, cardiovascular health, pulmonary function, and other pertinent medical indicators. Structured questionnaires and in-depth interviews were used to gather quantitative and qualitative data on patient experiences, satisfaction levels, and perceived health outcomes following prosthodontic procedures. Additionally, socio-economic factors such as income level, educational background, and occupational status were included to provide a more comprehensive demographic profile. Follow-up visits were conducted at intervals of <1 month, one to three months, and beyond three months after the intervention to monitor post-operative health, including symptoms and signs of respiratory infections. Clinical assessments included diagnostic tests such as sputum cultures and blood tests to confirm and analyze the presence of infections.

Statistical analysis

Statistical analysis was done by SPSS Statistics software (version 27; IBM Corp., Armonk, NY). This included employing descriptive statistics such as means, standard deviations (SDs), and frequencies to summarize participant demographics and outcomes. To evaluate correlations between prosthodontic procedures and the risk of chest infections, logistic regression analysis was used. The chi-square test was used to analyze the proportion of chest infections among the different intervention types. A paired t-test was run to analyze the distribution of respiratory health parameters before and after prosthodontic interventions. At p < 0.05, statistical significance was established.

Ethical approval

The institutional review board (IRB), Dental College, Heavy Industries Taxila Education City Institute of Medical Sciences (HITEC-IMS), Taxila, Pakistan (HITEC-IMS-IRB-03), granted ethical permission for this study, guaranteeing that ethical guidelines and patient privacy were followed throughout the investigation.

## Results

The following demographic traits were present in this research (Table 1), which included 130 participants. With a mean age of 52.4 years and a SD of 8.1 years, the age distribution revealed 33 people (25.38%) aged 18-39 years, 66 persons (50.77%) aged 40-59 years, and 31 individuals (23.85%) aged 60 years and beyond. The participants' gender was equally distributed; 65 (50.00%) identified as male, while the remaining participants identified as female. In terms of comorbidities, there were 19 individuals (14.62%), 31 participants (23.85%), and 43 participants (33.08%) who had diabetes and other medical disorders.

**Table 1:** Demographic characteristics of participants (n=130)

Characteristic	Category	Number of patients (n)/mean ± SD	Percentage (%)
Age group	18-39 years	33	25.38
	40-59 years	66	50.77
	60+ years	31	23.85
Age (years)	Mean ± SD	52.4 ± 8.1	-
Gender	Male	65	50.00
	Female	65	50.00
Comorbidities	Hypertension	43	33.08
	Diabetes	31	23.85
	Others	19	14.62

**Figure 1:**
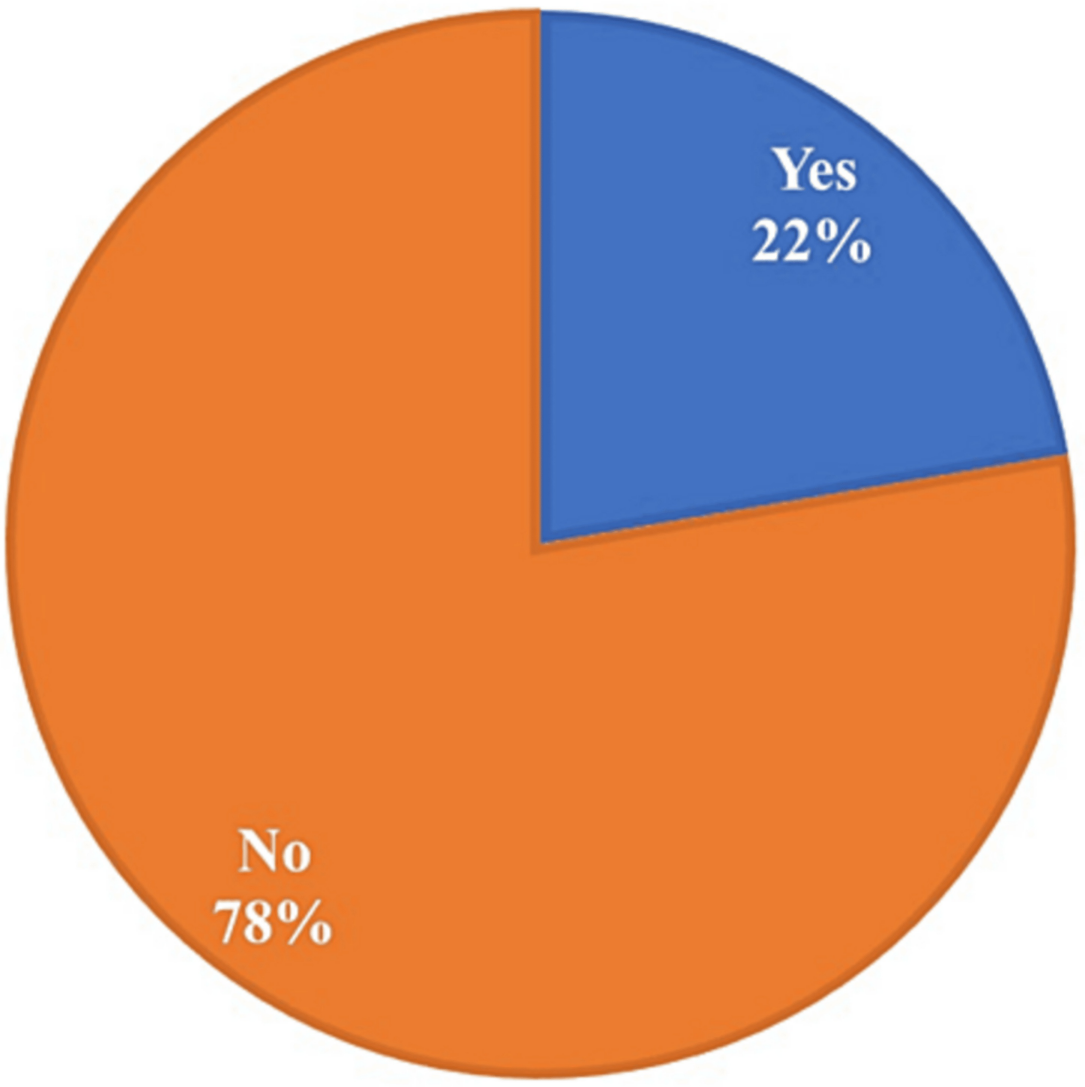
Distribution of chest infections among participants (n=130)

The distribution of chest infections among the 130 research participants is shown in Figure 1. Out of them, 29 individuals (22%) reported having chest infections, while the majority of participants (n=101, 78%), reported having no chest infections.

**Figure 2:**
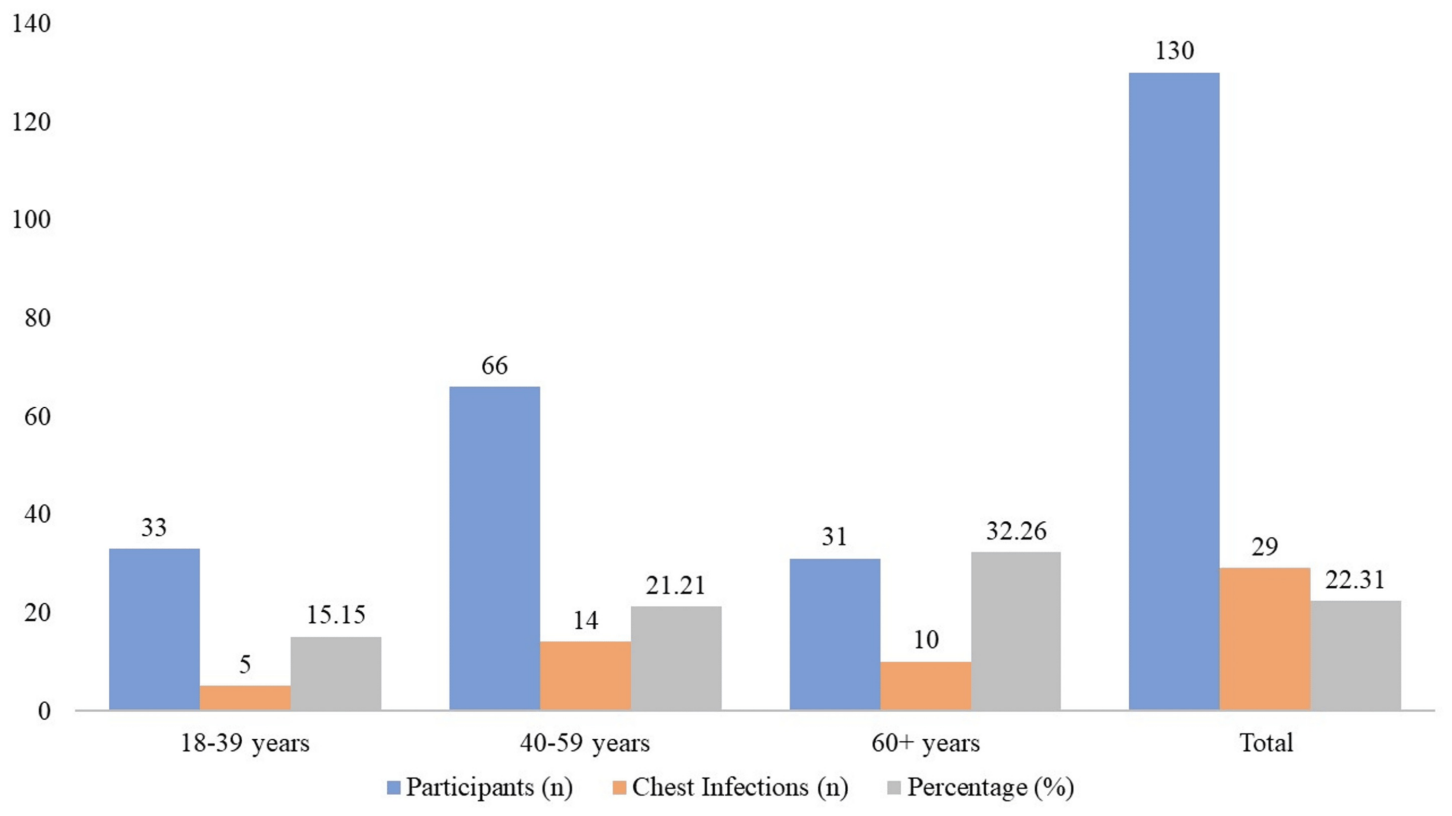
Comparison of chest infection rates by age group

The incidence of chest infections in various age groups after prosthodontic procedures is compared in Figure 2. Of the people in the 18-39 age range, five out of 33 (15.15%) had a chest infection. Ten out of 31 people (32.26%) in the 60+ age group and 14 out of 66 participants (21.21%) in the 40-59 age group reported having chest infections. A total of 29 occurrences of chest infections were reported among the 130 trial participants, yielding an overall infection rate of 22.31%.

**Figure 3:**
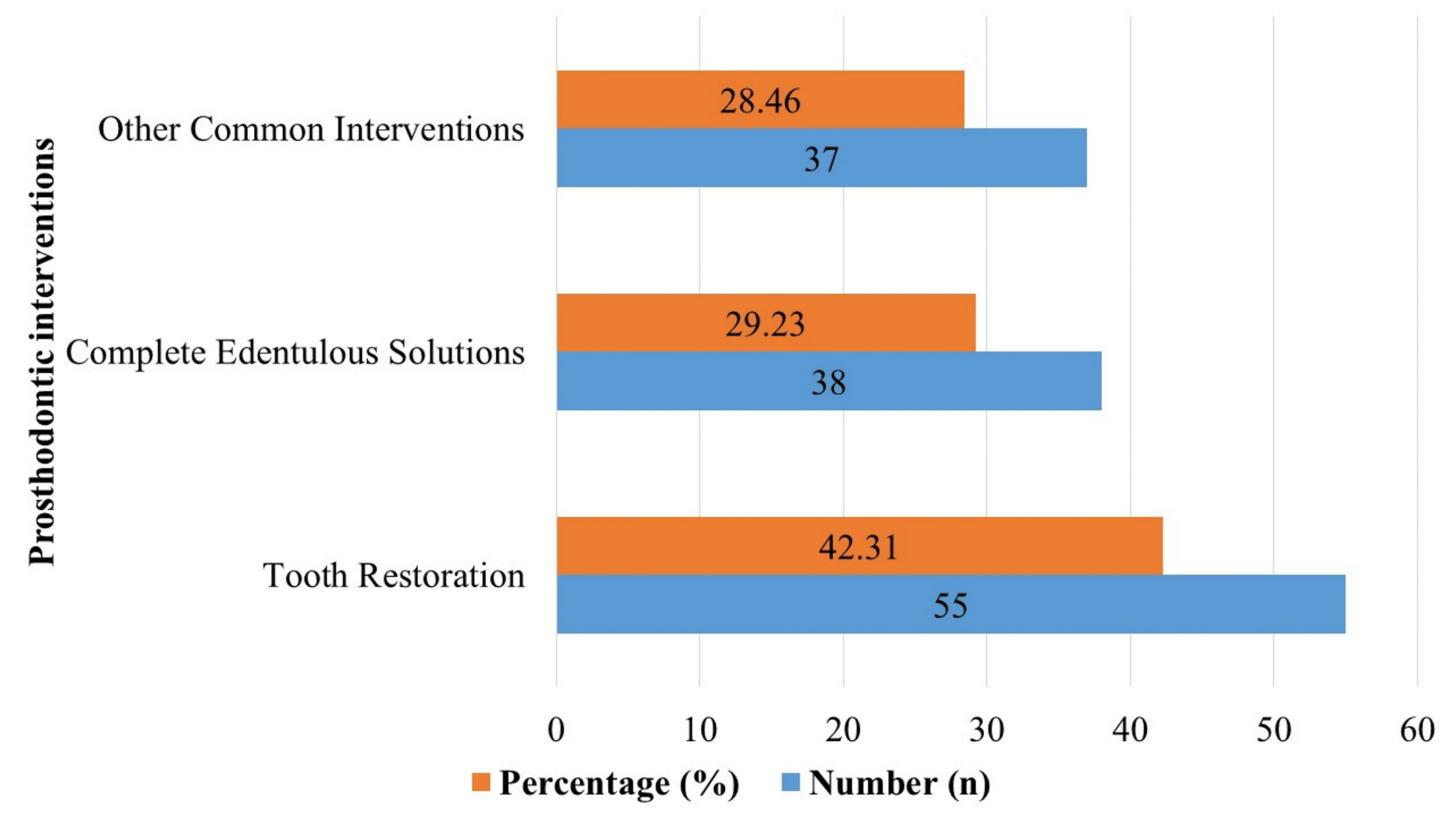
Distribution of prosthodontic interventions among 130 patients

The distribution of prosthodontic interventions among a sample of 130 patients is shown in Figure 3. With 55 instances, or 42.31% of the sample as a whole, tooth restoration was the most prevalent intervention type, according to the data. Next came complete edentulous solutions, with 38 instances total - 29.23% of the sample. Of the overall sample size, 28.46% consisted of 37 instances where other common interventions (bridgework, partial dentures, and implant-supported crowns) were detected.

The distribution of prosthodontic treatments and the findings from the logistic regression analysis pertaining to chest infections are shown in Table 2. Sixteen patients, or 55.17% of the sample, out of 55 instances of tooth restoration had chest infections; the odds ratio was 1.75, and the 95% confidence interval (CI) ranged from 1.12 to 2.63. Eight patients (27.59%) out of 38 instances with complete edentulous solutions experienced chest infections; this corresponded to an odds ratio of 2.21 (95% CI: 1.45, 3.18). An odds ratio of 1.46 (95% CI: 0.98, 2.21) was linked to five individuals (17.24%) who had chest infections as a consequence of other common interventions, which accounted for 37 cases. The chi-square test result (χ² = 3.15, df = 2, p = 0.207) indicates no statistically significant association between the type of intervention and the occurrence of chest infections. These results illustrate the odds ratios and incidence rates of chest infections for various prosthodontic procedures, offering information on possible correlations between these outcomes and respiratory health outcomes.

**Table 2:** Distribution of prosthodontic interventions and logistic regression analysis Df: Degrees of Freedom, x2: Chi-Square Statistic, P-value <0.05 is significant

Intervention type	Chest infection (n; %)	Odds ratio	95% CI	x^2^	df	P-value
Tooth restoration (n=55)	16 (55.17)	1.75	(1.12, 2.63)	3.15	2	0.207
Complete edentulous solutions (n=38)	8 (27.59)	2.21	(1.45, 3.18)			
Other common interventions (n= 37)	5 (17.24)	1.46	(0.98, 2.21)			

**Figure 4:**
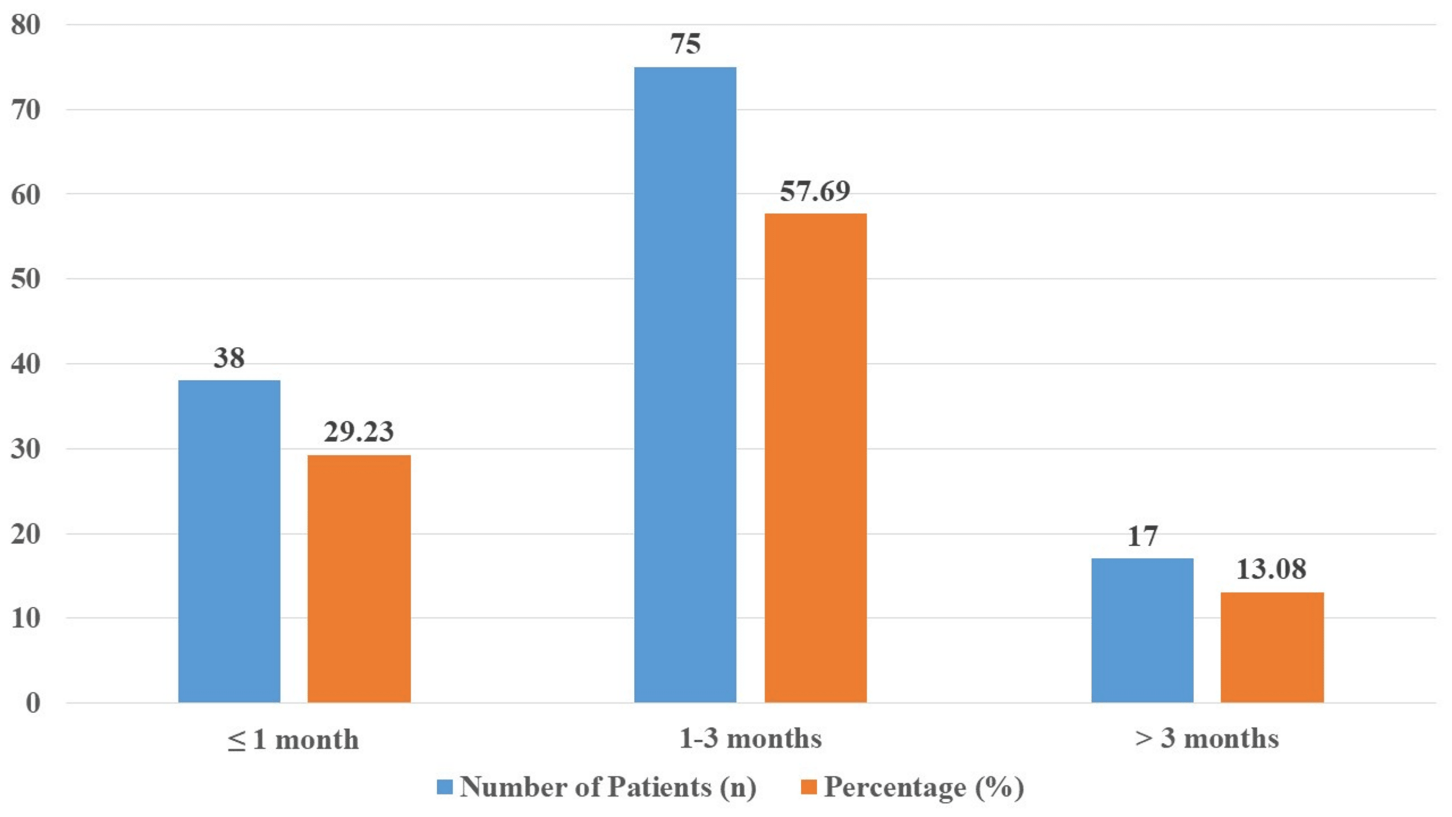
Distribution of follow-up visits timing among study participants

Data on the participants in the study's follow-up visits are shown in Figure 4. There were 130 participants in all, of whom 38 (29.23%) were planned for follow-up appointments within <1 month following treatment, 75 (57.69%) between one and three months, and 17 (13.08%) after more than three months.

Table 3 shows how the 130 research participants' respiratory health measures changed before and after prosthodontic procedures. Prior to the treatments, the subjects' average respiratory rate was 18.5 breaths per minute (± 2.1), their average oxygen saturation was 97.3% (± 1.5), and they coughed 2.7 times (± 0.9) on a daily basis. These values improved after the interventions: oxygen saturation increased to 98.1% (± 1.2), cough frequency decreased to 1.9 times per day (± 0.6), and respiratory rate decreased to 17.8 breaths per minute (± 1.8). The respiratory rate showed no significant change (p = 0.17), oxygen saturation increased significantly (p = 0.01), and cough frequency decreased highly significantly (p < 0.001).

**Table 3:** Distribution of respiratory health parameters before and after prosthodontic interventions

Parameter	Before intervention (mean ± SD)	After intervention (mean ± SD)	t-statistic	P-value
Respiratory rate (breaths/min)	18.5 ± 2.1	17.8 ± 1.8	1.41	0.17
Oxygen saturation (%)	97.3 ± 1.5	98.1 ± 1.2	-2.68	0.01
Cough frequency (times/day)	2.7 ± 0.9	1.9 ± 0.6	4.06	<0.001

## Discussion

The current research looked at 130 individuals' histories of chest infections after prosthodontic treatments. It found a significant incidence rate of 22.31% for chest infections following procedures. The possible effects of dental therapies on respiratory health outcomes are highlighted by this result. Chest infection rates after dental operations have been shown to fluctuate in the past; some studies have shown reduced frequencies [11,13]. Our findings are consistent with existing ones, indicating that while prosthodontic procedures are typically safe, respiratory problems need to be watched out for, especially in susceptible groups.

Our research examined several prosthodontic procedures and found varied degrees of correlation with chest infections. For example, a significant 55.17% incidence of chest infections was linked to dental restoration treatments (n=55), with an odds ratio of 1.75 (95% CI: 1.12, 2.63) resulting from this association. This discovery runs counter to research from other studies [14,15], which indicated reduced risk profiles for comparable therapies. The disparity highlights the need for more research into procedural variables and patient attributes that might account for variations in infection rates between studies.

In a similar vein, individuals (n=38) who had full edentulous solutions had an infection incidence of 27.59%, which was correlated with a higher odds ratio of 2.21 (95% CI: 1.45, 3.18). This conclusion is consistent with other research that has shown higher post-operative problems in patients involving total edentulism [16] and shows a possible increased risk linked to more comprehensive prosthodontic operations. Comprehending these subtleties is essential for maximizing patient outcomes in prosthodontic treatment and guiding clinical decision-making.

The context of our results is further provided by age-related differences in infection rates. The age group that showed the greatest prevalence of chest infections was 60 years and above (32.26%), followed by 40-59 years (21.21%) and 18-39 years (15.15%). The observed patterns in prior studies have consistently shown that older age is a substantial risk factor for post-operative problems after dental operations [7,17,18]. These age-dependent variations are consistent with those trends. These revelations highlight the significance of age-specific risk assessment and management techniques in prosthodontic treatment.

Improvements in respiratory rate, oxygen saturation, and frequency of coughing were found by analyzing changes in respiratory health markers after the intervention. These encouraging results point to the possible advantages of prosthodontic therapies for pulmonary function, which might be linked to better oral health and patient satisfaction after dental care. Analogous research has shown comparable improvements in respiratory parameters after dental treatments [19,20], supporting the idea that dental treatment might have a favorable impact on systemic health outcomes in addition to oral health.

Limitations of the study

Limitations of the study include several factors that may impact the generalizability and reliability of findings. Firstly, the study's single-center design at the Pakistan Institute of Medical Sciences may limit the applicability of results to broader populations with different demographic and health profiles. Secondly, the one-year duration may not capture long-term effects or seasonal variations in chest infections following prosthodontic procedures. Additionally, reliance on electronic health records for participant selection could introduce bias, as individuals with incomplete records or specific health conditions were excluded. Lastly, while statistical analysis was robust, the study's ability to establish causation between prosthodontic interventions and chest infections is constrained by its observational nature. Future research should consider multi-center designs and longer follow-up periods to address these limitations comprehensively.

## Conclusions

This research sheds important light on the intricate connection between respiratory health outcomes, especially chest infections, and prosthodontic therapies. The results of our study indicate a significant frequency of chest infections after dental procedures, underscoring the possible effects of dental treatments on overall health. The research emphasizes how important it is to keep an eye out for respiratory issues that may arise after prosthodontic operations, particularly in elderly patients and those receiving intensive dental care. This study adds to the body of knowledge guiding clinical practice by clarifying age-related changes and the risks associated with certain interventions. It also emphasizes the significance of customized patient care techniques in maximizing post-operative outcomes.

## Appendices

Questionnaire for evaluating the correlation between prosthodontic interventions and chest infections

Instructions for Participants

Please answer all questions to the best of your knowledge.

If you are unsure about any medical details, please consult your healthcare provider before completing the questionnaire.

Your responses will be kept confidential and used solely for research purposes to better understand the correlation between prosthodontic interventions and chest infections.

Section 1: demographic information

1. Age:

2. Gender:

Male

Female

Other (please specify)

3. Comorbidities:

Hypertension

Diabetes

Other (please specify)

None

Section 2: medical history and current health status

4. Do you have a history of respiratory illnesses?

Yes

No

5. Current Smoking Status:

Never smoked

Former smoker

Current smoker

6. Current Medications (if any): (Please list)

Section 3: prosthodontic procedures

7. Type of Prosthodontic Procedure:

Complete Edentulous Solutions (e.g., full dentures)

Tooth Restoration (e.g., crowns, bridges, implants)

Other (please specify)

8. Date of Prosthodontic Procedure: (DD/MM/YYYY)

9. Reason for Prosthodontic Procedure:

Functional (e.g., chewing, speaking)

Aesthetic

Both

Other (please specify)

Section 4: post-procedural health

10. Have you experienced any respiratory issues following the prosthodontic procedure?

 Yes

 No

11. If yes, what type of respiratory issues have you experienced? (Check all that apply)

 Coughing

 Shortness of breath

 Chest pain

 Fever

 Other (please specify)

12. How long after the procedure did you start experiencing respiratory issues?

 Within a week

 1-2 weeks

 2-4 weeks

 More than 4 weeks

13. Have you been diagnosed with a chest infection by a healthcare professional following the procedure?

 Yes

 No

Section 5: follow-up and healthcare

14. Have you had any follow-up visits with your dentist or healthcare provider since the procedure?

 Yes

 No

15. If yes, when was your last follow-up visit?

 Within 1 month after the procedure

 1-3 months after the procedure

 More than 3 months after the procedure

16. Were any additional treatments or medications prescribed during your follow-up visit?

 Yes

 No

17. If yes, please specify the treatments or medications:

Section 6: respiratory health parameters (pre and post procedure)

18. Respiratory rate

 Before procedure: ___ breaths per minute

 After procedure: ___ breaths per minute

19. Oxygen saturation

 Before procedure: ___%

 After procedure: ___%

20. Frequency of coughing:

 Before procedure: ___ times per day

 After procedure: ___ times per day

Section 7: Patient feedback and satisfaction

21. How satisfied are you with the outcome of your prosthodontic procedure?

 Very satisfied

 Satisfied

 Neutral

 Dissatisfied

 Very dissatisfied

22. Have you noticed any improvement in your overall respiratory health since the procedure?

 Significant improvement

 Some improvement

 No change

 Some deterioration

 Significant deterioration

23. Additional Comments or Concerns: (Please provide any other relevant information or concerns you may have about your prosthodontic procedure and its impact on your health.)

Thank you for cooperation!
